# Recent Applications of Ionic Liquids in Separation Technology

**DOI:** 10.3390/molecules15042405

**Published:** 2010-04-05

**Authors:** Dandan Han, Kyung Ho Row

**Affiliations:** Department of Chemical Engineering, Inha University, 253 Yonghyun-Dong, Nam-Ku, Incheon 402-751, Korea; E-Mail: hdd_216@hotmail.com (D.H.)

**Keywords:** ionic liquids, separation technology, supported membranes, mobile phase additive, surface-bonded stationary phase

## Abstract

Ionic liquids (ILs) have been applied in different areas of separation, such as ionic liquid supported membranes, as mobile phase additives and surface-bonded stationary phases in chromatography separations and as the extraction solvent in sample preparations, because they can be composed from various cations and anions that change the properties and phase behavior of liquids. Although the applications of ILs in separations are still in their early stages, the academic interest in ILs is increasing. An overview of the principle applications of ILs in separation technology is present in this work. Furthermore, the prospects of the ILs in separation techniques are discussed.

## 1. Introduction

Ionic liquids (ILs) are gaining widespread recognition as novel solvents in chemistry. Compared to classical organic solvents, ILs generally consist of bulky, nonsymmetrical organic cations such as imidazolium, pyrrolidinium, pyridinium, ammonium or phosphonium and numerous different inorganic or organic anions such as tetrafluoroborate and bromide anions. The unique properties of ILs, such as a negligible vapor pressure, good thermal stability, tunable viscosity and miscibility with water and organic solvents, as well as good extractability for various organic compounds and metal ions, mainly depend on their special structures. Replacing organic solvents currently used in different extraction processes with ILs can be considered a “hot” research topic. The non-flammable, non-volatile nature of ILs makes them an excellent choice for the development of safer processes. Furthermore, their polarity, hydrophobicity, viscosity and other chemical and physical properties can be selected by choosing the cationic or the anionic constituent. ILs are regarded as “designer solvents” because of this tunable nature, which increases their potential applications. 

ILs are widely considered as alternatives to classical organic solvents and as such have been applied in many fields such as organic synthesis, electrochemistry, liquid phase extraction, catalysis for clean technology and polymerization processes [1,2,3,4,5]. Accordingly, their application in separation technology has attracted great attention. Alkylimidazolium-based ILs have been used as a stable stationary phase for gas chromatography [6,7]. These ILs exhibit an unusual selectivity with a “dual nature”, where they separate polar compounds as if they were polar stationary phases and non-polar compounds, which suggests that ILs might be useful multi-modal media in chromatographic separations. ILs can be used as mobile phase additives in reversed-phase chromatography when they are mixed with other low viscosity solvents [8,9]. ILs have been used as electrolyte additives, running buffer modifiers and supported coatings on the capillary walls in capillary electrophoresis (CE) [10,11]. In recent years, many studies have been conducted by our group on the applications of ILs in separations [12,13,14,15,16,17,18,19,20,21,22,23]. In the review, the physicochemical properties of ILs are introduced and a brief overview of the applications of ILs in separation technology is provided, with a special focus on their use as extraction solvents in sample preparations, ionic liquid supported membranes, mobile phase additives and surface-bonded stationary phases in chromatographic separations.

## 2. General Characteristics of ILs 

ILs are usually composed of large asymmetric organic cations and inorganic or organic anions. They exhibit unique properties compared to other solvents. First, ILs are generally colorless liquids with relatively high viscosities. Second, they exhibit very low vapor pressures under ambient conditions and thus are effectively non-volatile. Third, ILs are good solvents for a broad spectrum of inorganic, organic and polymeric materials and are immiscible with numerous organic solvents. Some properties, such as the thermal stability and miscibility, mainly depend on the anion, while others, such as the viscosity, surface tension and density, depend on the length of the alkyl chain in the cation and/or shape or symmetry [24,25]. The most commonly employed IL anions are polyatomic inorganic species, such as PF_6_^-^ and BF_4_^-^. The most prominent cations are pyridinium and an imidazolium ring species with one or more alkyl groups attached to the nitrogen or carbon atoms. The physical properties of some ionic liquids are presented in Table 1. In addition to the interactions that exist in conventional organic solvents (hydrogen bonding, dipole–dipole and van der Waals interactions). ILs also have ionic interactions (mutual electrostatic attraction or repulsion of charged particles), which makes them very miscible with polar substances. At the same time, the presence of the alkyl chain on the cation determines the solubility of the ILs in less polar fluids. Hydrogen bonding is thought to exist between the oxygen or halide atoms on the anion and the hydrogen atoms on the imidazolium or pyridinium ring of the cation in ILs. The properties of the ILs can significantly change by varying the length of the alkyl groups that are incorporated into the cation and the types of anions. For example, for the 1-alkyl-3-alkylimadazolium cation, replacing the (PF_6_^−^) anion with (BF_4_^−^) dramatically increases the solubility of the IL in water, whereas replacing the anion with the (Tf_2_N^−^) ion decreases the water solubility. The change in the length of the 1-alkyl chain from 1 to 9 on 1-alkyl-3-methyl-imadazolium hexafluorophosphate [CnMIm][PF_6_] can make the usually soluble ILs very immiscible in water. 

## 3. Applications of ILs in Separation Technology 

### 3.1. ILs as mobile phase additives in liquid chromatography 

#### 3.1.1. Mechanism of action on separation of ILs as additives 

Basic compounds most often bear positively charged amine groups in low pH mobile phases. Consequently these compounds are retained by a combination of electrical (charge-charge) and hydrophobic interactions with the stationary phase and the ions of the mobile phase. They are not easily separated in RPLC with silica-based stationary phases because of the interaction between the cationic sites of the compounds and the anionic silanols of the stationary phase. In the aqueous mobile phases, the charges interactions are usually stronger and slower than the hydrophobic interactions, which produces peak tailing and long retention times. ILs can be used as mobile phase additives in reversed-phase chromatography when mixed with other solvents such as methanol and acetonitrile. The addition of ionic liquids as the mobile phase in HPLC decreases the band tailing, reduces the band broadening, and improves the resolution. The probably mechanism of interactions can be described as follows. The cations in ILs can interact and compete with silanol groups (specific electrostatic interactions) on the alkyl silica base surface with the polar group of the analytes. At the same time, the nonpolar alkyl groups of the stationary phase can interact with different alkyl groups of the heterocyclic ring or quaternary cation (unspecific type of interactions and hydrophobic interactions) [26,27]. Therefore, this phenomenon can efficiently shield the residual silanols and improve the peak shapes, while also reduce the chromatographic retention times of the analytes. The relationship between the concentration of ILs modifier and the chromatographic retention may be qualified using the following factors. The significant addition of ILs modifier to a mobile phase leads to competition between IL cations and the polar groups of analytes for the polar silanol groups on an alkyl silica surface, resulting in decreases in the retention times of analytes. The retention of nonpolar analytes will also be reduced, as the ILs modifier also disables the alkyl groups of the stationary phase, which leads to a sharp decrease in the possibility of dispersion interactions between the nonpolar analytes and the alkyl groups of the stationary phase.

With a further increase in the concentration of an ILs modifier, cations interact with the silanol groups through electrostatic interactions, producing a weak bilayer electronic structure, which repulses basic sorbates, and interacts with the alkyl groups through hydrophobic and non-specific interactions, so the retention of analytes decreases under due to these repulsive and hydrophobic interactions. If the concentrations of the ILs are slightly increased, their cation interactions with the silanol groups on the alkyl silica surface due to specific interactions or with the alkyl groups due to hydrophobic and nonspecific interactions gradually strengthen, resulting in an increase in the carbon content of the stationary phase; thus, the retention times of substances are increased. 

#### 3.1.2. Application of ILs as mobile phase additive 

Table 2 lists the applications of ILs as mobile phase additives [12,13,14,15,16,28,29,30,31,32,33,34,35,36,37]. Several groups have reported using ILs as mobile phase modifiers to improve liquid chromatography separations. The first time ILs were used as mobile phase additives was to study the chromatographic behavior of ephedrines and catecholamines [26]. The addition of 2.0–50.0 mM IL to the aqueous mobile phases improved the basic compound peak shapes, but these improvements were associated with the changes in the retention factors. A wide variety of ILs was investigated, and a model involving ion-pairing and IL layer that was adsorbed on the C_18_ surface was proposed. The result showed that the IL addition could decrease the band tailing, reduce the band broadening, and improve the resolution. Kaliszan *et al*. reported a significant improvement on the basic compound peak shape that was obtained after adding small amounts of ILs to the mobile phases [27]. The ILs had better silanol blocking activity than classical quaternary amines. However, they also observed that the improvements in peak shape were associated with significant changes in the retention factors. These retention factors can increase or decrease depending on the IL used. ILs behave as regular salts when they are used as additives for aqueous solutions at low concentrations. Their specific properties, such as their low melting point, high thermal stability and extremely low vapor pressure, are lost and/or not important. As salts, the ILs exhibit a dual nature. They are obviously made up of cations associated with an equal amount of anions and thus, both species can affect the chromatographic results.

Analyses of some nucleic and amino acids and the interaction mechanism of ILs have also been reported. In the work by Row *et al*. [13], the effects of the concentration of the IL 1-butyl-3-methyl-imidazolium [BMIM][BF_4_] as a mobile phase modifier on the retention of the nucleic (cytosine, cytidine, and thymine) and amino acids (N-CBZ-D-phenylalanine and D-tryptophan) were studied. The IL content affected the resolution between the D-tryptophan and N-CBZ-D-phenylalanine in the mobile phase. The resolution was improved with increasing IL concentration. The chromatograms that were generated using the IL in the mobile phase generally exhibited more symmetrical peaks and faster analysis times. This experiment also showed that the shape of the peaks (asymmetry and peak tailing parameters) was improved using the [BMIM][BF_4_] IL as an additive in the mobile phase. 

Other researchers have used different ILs as mobile phase modifiers in order to study the effects of the IL concentration, different alkyl imidazolium groups and different cation counterion on the analyte retention. In this work, the retention factor depended on the hydrophobic nature or chaotropic character of the IL anions. In a similar study [38], the researchers separated some amines, including benzidine, benzylamine, *N*-ethylaniline and *N*,*N*-dimethylaniline, using the ILs ([EMIM][BF_4_]), 1-butyl-3-methylimidazolium1-tetrafluoroborate1-([BMIM][BF_4_]), 1-hexanyl-3-methylimidazolium tetrafluoro-borate ([HMIM][BF_4_]) and 1-butyl-3-methyl-imidazolium bromide ([BMIM][Br]) as the mobile phase additives, respectively. Not as much of the amines were retained when the ILs were added and most of their retention factors decreased with increasing IL concentration. All of the *k* values of amines remarkedly decreased with increasing alkyl length of the imidazolium cation from ethyl, through butyl, to hexyl. 

ILs have not only been used in reversed-phase HPLC but also in normal HPLC. Marszałł [39] used ILs as the mobile phase additives in order to demonstrate the distinct differences in the retention and selectivity for both normal and reversed-phase HPLC. The four tested ILs exhibited very similar trends in both the normal and reversed-phase LC systems. However, the differences in the retention of the individual analytes in the specific LC systems caused practically useful changes in the separation of the basic analytes. The silanol-suppressing potency of ILs strongly surpassed that of the standard alkylamine additives. The use of ILs to replace the less effective and environmentally harmful alkylamine additives in LC improved the separation efficiency and laboratory safety.

The separation of chiral compounds has been of great importance in both research and industry, particularly in the pharmaceutical industry. The search for novel chiral selectors is an open field that still attracting the creative efforts of researchers all over the world. Yuan *et al*. [40] used the chiral IL (R)-N,N,N-trimethyl-2-aminobutanol-bis(trifluoromethanesulfon)imidate as a chiral selector in chromatography to separate eight racemates for the first time and obtained a better resolution than without the chiral IL. Their work indicates that the use of chiral ILs could soon become very attractive as a new chiral selector in the chromatography. These investigations showed that ILs could play multiple roles, such as blocking residual silanols groups, modifying the stationary phase, and acting as active ion-pairing agents as mobile phase additives. If the basic compounds are polar and lightly retained, a polar IL additive with a strongly chaotropic anion is recommended. With less polar and hydrophobic amines, a less polar IL additive with a cosmotropic anion might be a good choice. 

### 3.2. ILs used as extraction solvents in sample preparation 

#### 3.2.1. Extraction mechanisms 

Some ILs are suitable for conventional liquid-liquid extraction because of their immiscibility with water (which allows formation of biphasic systems) as well as the high solubility of the organic species in them. The design of safe and environmentally benign separation processes plays an increasingly important role in the development of extraction technology.

Huddleston *et al*. [41] studied the partition coefficients between the ILs and water and compared the results with the octanol-water partition coefficient. The results showed that these two kinds of coefficient exhibited a good linear relationship, and the distribution coefficient was higher for the uncharged form. Later, the ionic liquid/water and ionic liquid/heptane distribution coefficients of a set of 40 compounds with various functionalities, including organic acids, organic bases, amino acids, antioxidants, and neutral compounds, were measured using liquid chromatography by Armstrong’s group [42]. Marked differences in the partitioning behavior of basic, acidic, and neutral compounds were observed. This study has indicated a lower basicity of the ionic liquid phase compared to octanol. Acidic solutes have distribution coefficients lower than their distribution coefficients in octanol. The opposite is true for aminoaromatic compounds. These results could probably be attributed to the lower basicity of the ILs compared to octanol. In general, for ionizable compounds transfer from the aqueous phase to the room temperature ionic liquid is more efficient for the neutral form of the compound. Adjustment of the pH of the aqueous phase is an effective means of adjusting selectivity for extraction by ILs, as is the case for non-ionic solvents. 

#### 3.2.2. Extraction of organic contaminants and metal contaminants

Table 3 summarizes some representative examples of IL-based extractions of metal ions and organic contaminants in environmental samples, such as water, soil and so on [43,44,45,46,47,48,49,50,51,52,53,54,55,56,57,58,59,60,61,62,63,64].

Vidal *et al.* [65] evaluated a series of [CnMIM][PF_6_] and [CnMIM][BF_4_] ionic liquids for the extraction of phenol, tyrosol and *p*-hydroxybenzoic acid from aqueous solution. A near quantitative extraction of the three phenols was obtained using [OMIM][BF_4_] (the results were similar to those observed using *n*-octanol). Fan *et al.* [66] reported similar results for the extraction of phenol, bisphenol A, pentachlorophenol, 4-octylphenol, and 4-nonylphenol for the same series of ionic liquids. The increase in extraction efficiency for ILs as the length of the alkyl chain attached to the cation increased was assigned to the considerable importance of solute hydrophobicity on the extraction mechanism. The higher distribution constants observed for ILs containing the [BF_4_]^-^ anion compared with the [PF_6_]^-^ anion was assigned to the stronger hydrogen-bonding interactions of the phenols with the [BF_4_]^-^ anion. The ionic liquids were more than 10-fold more efficient at extracting the phenols from water than dichloromethane.

Dispersive liquid-phase microextraction takes advantage of the low solubility of the extraction solvent, in this case ILs such as [HMIM][PF_6_] to be dispersed throughout a larger sample (aqueous) volume assisted by a disperser solvent, and subsequently recovered from solution as a discrete drop. Liu *et al.* [43] first used IL based dispersive liquid-phase microextraction (IL-DLPME) of polycyclic aromatic hydrocarbons (PAHs) from water. A mixture of 0.052 g [HMIM][PF_6_] and 0.50 mL methanol (disperser solvent) was quickly injected into a sample solution with a 1 mL syringe. A cloudy solution was quickly formed as the fine droplets dispersed the immiscible extraction solvent in the aqueous sample which greatly enlarged the contact area between the extraction solvent and aqueous phase. The analytes in aqueous sample were extracted into the fine ionic liquid droplets at this step. Then the water–methanol–[HMIM][PF_6_] mixture was centrifuged at 4,000 rpm for 10.0 min. After this process, the dispersive particles of ionic liquid phase were sedimented at the bottom of a conical test tube. The upper aqueous phase was removed with a syringe, and the IL phase (about 19 μL) was dissolved in 50 μL methanol and 10 μL was injected into the HPLC system for analysis. The enrichment factor ranged from 10 to 200, which is about three times that obtained with 1-octanol. Using a similar protocol, chlorobenzenes, phenols, dichlorodiphenyltrichloroethane and its metabolites in a water sample were preconcentrated. A later study showed that many organic pollutants, including BTEX (benzene, toluene, ethylbenzene, and xylene), PAHs, phenols, aromatic amines, herbicides, and organomecury compounds can be preconcentrated by this method [43,45,46,47,48]. The advantage of dispersive methods is the greater surface area provided by the dispersed or dissolved extraction solvent, which enhances the rate of analyte transfer to the extraction solvent.

Hollow fiber based-liquid phase microextraction (HF-LPME) using ionic liquids also has been used in extraction. The extraction solvent is immobilized within the pores of the membrane and forms a liquid barrier between the donor phase (sample solution) and acceptor phase (injection solvent). For aqueous donor phases the room temperature ionic liquids [OMIM][PF_6_] was shown to be suitable solvents for the extraction of chlorophenols into a basic buffer. Limits of detection for the chlorophenols were in the range of 0.5–2.5 µg/L, which is suitable for analysis of typical environmental samples [67]. 

Since ILs are a group of thermally stable and nonvolatile liquids, they are very suitable for high-temperature headspace single-drop microextraction (HS-SDME) of volatile and semi-volatile compounds. Peng *et al*. [68] used this method to extract four anilines in environmental water samples at temperatures as high as 90 ºC. The high temperature enhanced the volatilization of analytes into the head space and thus improved the enrichment factor. Recently, Zhou *et al*. [69] developed a temperature controlled IL dispersive liquid phase microextraction technique for extracting pyrethroid pesticides in water samples. A homogeneous phase of IL and water was obtained by heating, while cooling of the homogeneous liquid mixture produced phase separation due to the decreased solubility. Briefly, 45 μL of [HMIM] [PF_6_] was added to about 10 mL of a water sample, and this was then heated to 70 ºC, which enabled the IL to completely dissolve in water. After the extraction, the system was cooled down and phase separation of the IL from water was realized by centrifuge. The analyte containing IL was then diluted for HPLC analysis. Some amino-acid based ILs [70] that have lower critical solution temperatures have the potential for use in sample preparation.

Extraction of metal ions from polluted environmental sample is another main use of ILs. Visser *et al*. studied the extraction of Na^+^, Cs^+^, and Sr^2+^ from aqueous solution into [CnMIM][PF_6_], (n = 4, 6, 8) by crown ethers [71]. Hg^2+^, Cd^2+^ [72,73], and Am^3+^ [74] were successfully extracted from the aqueous phase into task-specific ILs, or a mixture of the task-specific ILs and a less expensive conventional IL. Ag+, Hg^2+^, Cu^2+^, Pb^2+^, Cd^2+^ and Zn^2+^ were successfully extracted into [BMIM][PF_6_] by employing dithizone as a chelator to form neutral metal-dithizone complexes [75]. It was found that the extraction efficiency of IL is higher than that of chloroform at low pH. Furthermore, metal ions can be extracted from aqueous phase into [BMIM][PF_6_] and then back extracted into aqueous phase with high recovery by manipulating the pH value of the extraction system.

Recently, it was reported that Hg^2+^can be extracted into [CnMIM][PF_6_] (n = 4, 6, 8) without the need for any complexing reagent [76]. Heating the ILs to a moderate temperature (60 ºC for [OMIM][PF_6_]) accelerates the extraction process, and up to 90–100% of HgCl_2_ in 0.15 M acetate buffer (pH 4.68) can be transferred into ILs over timescales ranging from several hours to 100 h. 

#### 3.2.3. Extraction of bioactive compounds in natural plant

As indicated in Table 4, the extraction of bioactive compounds from natural plants using ILs shows great promise. Liquid-liquid extraction, liquid-phase microextraction(LPME), solid-phase micro-extraction (SPME) and aqueous two-phase systems extraction with ILs could alleviate environmental pollution and improve the selectivity and the extraction yields of interesting compounds in the sample pretreatment processes in comparison to conventional organic solvents [77,78,79,80,81,82,83,84,85,86,87,88,89]. Microwave-assisted extraction (MAE) is an attractive and rapid sample preparation technique, and ILs could potentially be applied as solvents in the MAE of various useful substances from solid samples because ILs can efficiently absorb microwave energy. Recently, 1-*n*-butyl-3-methylimidazolium-based ionic liquid aqueous solutions were investigated as solvents in the extraction of *trans*-resveratrol from *Rhizma polygoni Cuspidati* [77] and alkaloids from medicinal plants [78], indicating that ILs could potentially be applied to the MAE of useful substances from medicinal plants. Many medicinal plants contain various bioactive compounds, such as polyphenolic compounds, nitrogen compounds, vitamins, terpenoids and some other endogenous metabolites, and have been used in pharmaceutical and dietary therapy for several millennia. Cao *et al.* [79] developed an ionic liquid-based ultrasonic-assisted extraction (ILUAE) method for separating piperine from white pepper powder. A series of 1-alkyl-3-methylimidazolium ionic liquids with different alkyl chain and anion composition were evaluated with respect to their extraction efficiency. The results indicated that both the characteristics of the anions and cations remarkably affected the extraction efficiency. The optimized approach exhibited the highest extraction efficiency (from 1.950% to 3.577%) within the shortest extraction time (from 2 h to 30 min) compared to the conventional heat-reflux extraction (HRE) and regular ultrasonic assistant extraction (UAE).

### 3.3. ILs used as surface-bonded stationary phases 

#### 3.3.1. Mechanism of action of ILs as surface-bonded stationary phases

In recent years, different separation media have been investigated and developed for chromatographic separations. Generally, normal-phase HPLC columns with silica sorbents and reverse-phase HPLC with C_18_ as the stationary phase are the most widely used techniques. Many studies have placed an emphasis on improving the different stationary phases with various structures in order to improve the selectivity of HPLC. 

Ionic liquid-modified silica, which consists of bulky organic cations that are combined with inorganic or organic anions has recently been developed as a new sorbent material. This media is receiving a lot of attention because of its excellent properties and potential applications in many fields of analytical chemistry. Ionic liquid-modified polymers are another recently developed sorbent material. Functional porous polymers have been used as the stationary phase in HPLC separation in order to improve the selectivity, but their main limitation is the low column efficiency. These polymers can be modified using ILs as better alternatives to the conventional stationary phases in order to increase the column efficiency.

#### 3.3.2. Applications of ILs as surface-bonded stationary phases

Several new IL bonded-surface stationary phases have been synthesized and the columns obtained were used to separate a variety of solutes for linear solvation energy relationship (LSER) studies [90]. The 1-butyl-3-heptylimidazolium bromide stationary phase was similar to conventional phenyl-based stationary phases under reversed-phase conditions for the separation of a group of neutral aromatic solutes [91]. Despite the complexity of the novel stationary phase, the excellent correlation for the global fit between the experimental and calculated retentions across the mobile phase composition range in these studies, supported the use of the LSER model to describe the retention for this limited set of solutes under the normal phase conditions.

Table 5 summarizes some applications of ILs as surface-bonded stationary phases [92,93,94,95,96,97,98,99,100,101,102]. A new anion-exchange stationary phase based on *N*-methylimidazolium was made through the reaction of activated silica with 3-chloropropyltrimethoxysilane and subsequently with *N-*methylimidazole [103]. Familiar inorganic anions, organic anions and some other organic compounds were successfully separated using this new stationary phass, and a high column efficiency and satisfactory resolution were obtained. The phase based on *N*-methylimidazolium was an anion-exchange phase, but it possessed multi-modal retention properties because it also had reversed-phase interactions and a hydrogen bonding interactions. This study showed the new phase was capable of separating some biological samples such as amino acids and proteins. 

Another successful use of the IL bonded-surface stationary phase was the separation of different analytes. For example, Qiu *et al.* [104] synthesized a new zwitterionic stationary phase based on silica bonded with 1-alkyl-3-(propyl-3-sulfonate) imidazolium. The materials were confirmed and evaluated using elemental analysis, thermogravimetric analysis and X-ray photoelectron spectroscopy. Potassium and calcium were simultaneously separated with several common inorganic anions, including iodate, chloride, bromide, nitrate and iodide in the phase. Liu *et al.* [105] synthesized a new and effective, IL-based stationary phase that was applied to the separation of ephedrines using HPLC. The separation results indicated that the stationary phase exhibited a high efficiency and reproducibility. The effective separation was attributed to the electrostatic and ion-exchange interactions between the solutes and the stationary phase. Moreover, the free silanols on the surface of the silica were effectively masked by the immobilized IL, resulting in the decrease of in non-specific absorption.

More recently, two new stationary phases based on [PMIM]Br and [BMIM]Br ILs were studied for separation of a group of organic acids [106]. The hydrophobic and ion-exchange interactions seemed to be the major factors that contributed to the retention. One of the main advantages of the surface-immobilized ILs stationary phases was that the effective separation was achieved using aqueous mobile phases with very little to no organic solvent. 

The surface-immobilized ILs also can be used as solid phase extraction sorbent. A disposable IL-coated headspace SPME fiber was created in order to determine BETXs in paints by Liu *et al*. [107]. This SPME fiber has some advantages over commercial SPME fibers, including a much lower cost per fiber, comparable reproducibility (RSD <11%), and elimination of carryover effects. This method was later improved by Hsieh *et al*. [108] in order to determine trace PAHs in water sample. Nafion was coated onto the fiber prior to the IL coating, which enhanced the amount and stability of the IL film adsorbed onto the fiber. Recently, Li *et al.* [109] used silica coated with [CnMIM]Br (n = 8, 12) as a sorbent for the solid-phase extraction of phthalates in environmental water samples. Five target analytes were quantitatively extracted with enrichment factors as high as 600. The negligible volatility of IL prohibited the direct coupling of IL-based LPME with GC. This is because the injection of ILs into GC could contaminate the GC column. Tian *et al*. used ionic liquid-modified silica sorbents as a special sorbent in solid-phase extraction process to isolation of bioactive compounds from natural plant [20,21]. Compared to other C_18_, silica sorbents, IL surface-bonded silica of the polymer showed higher affinity and better extraction efficiency. 

### 3.4. Preparation of supported IL membranes 

#### 3.4.1. IL membranes for the selective transport of organic compounds

Porous and nonporous membrane processes can be used to accomplish this separation. In the porous membranes, the selectivity is based on the size and mass of the molecules that are being separated. The selectivity can be significantly enhanced using special liquids that immobilized in the pores of the membranes (supported ionic liquid membranes, SILMs). The SILM system includes a feed solution, a solvent or solvent/carrier that was immobilized in the porous structure of a polymeric or ceramic membrane, and a receiving solution. The analyte that is dissolved in the feed solution goes through the membrane and is enriched in the receiving solution. The organic solvent supported liquid membrane is not stable because of the loss of the immobilized solvent via evaporation and its dissolution in the feed or the receiving solution. The unique properties of ILs, such as their negligible vapor pressure and immiscibility with water and some organic solvents, can be used to overcome these problems. Another important advantage of using SILMs in the gas separation is that the solvent is not lost through volatilization, and therefore, very stable membranes are obtained. A previously study proved that supported liquid membranes (SLMs) that were immobilized with RTILs were stable for gas-gas separations [110].

#### 3.4.2. Gas separation by supported ILs membranes

Recently, the use of ILs for gas separation processes has attracted much attention because of the high solubility of different gaseous species in ionic liquids. The preparation of SILMs has also been reported for the selective separation of different organic or gaseous compounds. The CO_2_ separation from gas streams is a worldwide topic of interest for decreasing the global warming causing gas emissions. Additionally research has focused on the refinement of biogas for the feed-in of biologically produced methane into conventional natural gas distribution networks.

Sulfur compounds are interesting contaminants in many industrial gas streams. H_2_S is a toxic and corrosive substance that is found in nearly all natural gas sources and synthesis gas and biogas streams. SO_2_ emissions are generated wherever fossil fuels are combusted. Other sulfur compounds, like tetrahydrothiophene (THT), are added to natural gas as odorants, making it impossible to directly use natural gas in peripheral catalytic processes like home-based fuel cells for heat and electricity generation because of catalyst poisoning. This present investigation continuously separated gas compounds like CO_2_, H_2_S, THT and SO_2_ from N_2_ or CH_4_ using supported ionic liquid membranes. Table 6 provides some recent examples of this separation [111,112,113,114,115,116,117,118,119,120,121,122,123]. Therefore, an adequate porous support material, e.g. a polymer film, was coated with ionic liquids and was successfully tested for the continuous separation of CO_2_ and the sulfur compounds from different gas mixtures. The influence of the support properties, the ionic liquid and the gas flow on the achievable degree of separation, i.e. the permeability and the selectivity, was studied. The results indicated that the selectivity and permeability were competitive compared to the industrial relevant processes based on polymer membranes. The task specific synthesis of ILs could potential exceed the conventional polymer membranes efficiencies because of the solubility and diffusivity properties of the ILs.

## 4. Perspectives

ILs have many fascinating properties that make them different from conventional solvents. The use of ILs is opening new opportunities in different areas of separation science, with new many applications. Further applications in separations are related to the pharmaceutical, biomedical, environmental and other industries. ILs have been explored in separations for extraction, supported liquid membranes, as additives and as stationary phases in chromatography.

The use of ILs as additives in chromatography exhibits great advantages compared to other additives. However, the applications of user designed ILs are not well developed. ILs with different cations and anions must be synthesized to separate various target compounds with different properties. The economy of using ILs as additives should be considered. The economic use of ILs could be achieved by recovering the ILs from the chromatography waste.

IL-modified materials have proven to be an important new type of stationary phases. These IL stationary phases could be commercialized after some improvements involving the separation selectivity, thermal stability, immobilization bonding stability and so on. The stability of the stationary phase is considered an emerging handicap for commercially applications. Additionally, the range of separation compounds must be broadened from normal chemicals to bioactive compounds. More attentions must be paid to chiral IL-based stationary phases because of the reasonable efficiency of the enantioresolution of the chiral analytes. 

ILs have been advertised as green solvents due to their nonvolatility. However, the environmental benefits of ILs need to be carefully considered. Not all ILs are safe and nontoxic, especially during their synthesis. Therefore, the development of real green ILs for extraction is still a challenge.

## Figures and Tables

**Table 1:** Physical properties of ionic liquids in separation technology.

Chemical formula		Abbreviation	Melting point, ºC	Density (g mL^-1^), 25 ºC	Viscosity (cP), 25 ºC	Molecular weight
Cation	Anion					
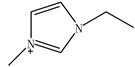	[BF_4_]^-^	[EMIM][BF_4_]	6	1.248	66	197.8
	[PF_6_]^-^	[EMIM][PF_6_]	58-62	1.373	450	256.13
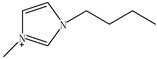	[BF_4_]^-^	[BMIM][BF_4_]	-82	1.208	233	225.80
	[PF_6_]^-^	[BMIM][PF_6_]	10	1.373	400	284.18
	[Br]^-^	[BMIM]Br	60	1.134	Solid	218.9
	[Cl]^-^	[BMIM]Cl	89	1.120	Solid	146.50
	[CF_3_SO_3_]^-^	[BMIM][CF_3_SO_3_]	16	1.290	90	260.0
	[(CF_3_SO_2_)_2_N]^-^	[BMIM] [(CF_3_SO_2_)_2_N]	-4	1.420	52	487.9
	[NTfO_2_]^-^	[BMIM] [NTfO_2_]	-8	1.404	48	433.0
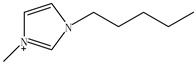	[BF_4_]^-^	[AMIM][BF_4_]	-88	1.231	321	240.02
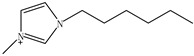	[BF_4_]^-^	[HMIM][BF_4_]	-82	1.075	211	254.08
	[PF_6_]^-^	[HMIM][PF_6_]	-61	1.304	800	312.00
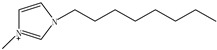	[BF_4_]^-^	[OMIM][BF_4_]	-79	1.11	440	281.8
	[Cl]^-^	[OMIM][Cl]	0	1.000	16,000	230.50
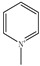	[NTfO_2_]^-^	[MPPyr] [NTfO_2_]^-^	0	1.44	39	416
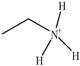	[HCOO]^-^	BAF	-10	0.99	11.5	91
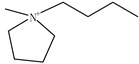	[NTfO_2_]^-^	[BMPyrrol] [NTfO_2_]	-50	1.4	71	422

**Table 2:** Application of ionic liquid as the mobile phase additives.

Target compounds	Mobile phase	Ionic liquid additive	Ref.
Nucleotides	Methanol /water (10:90, v/v)	13.0 mM of [BMIM][BF_4_]	[12]
Amino acid and Nucleic acid	Methanol /water (65:35, v/v)	2.0 mM of [BMIM][BF_4_]	[13]
*N*-CBZ-D-Phenylalanine, and D-tryptophan	Acetonitrile/water (50:50, v/v)	10.0 mM of [BMIM][BF_4_]	[14]
Amino benzoic acids isomers	Methanol /water (25:75, v/v)	13.0 mM of [BMIM][BF_4_]	[15]
Polar compounds	Methanol /water (65:35, v/v)	1.86 mM of [EMIM][MS]	[16]
Matrine, oxymatrine, sophoridine, Sophocarpine	Methanol /water (45:55, v/v) at pH 3.0	0.1 mM of [BMIM][BF_4_]	[28]
Antidepressants	NaH_2_PO_4_/ acetonitrile / methanol (70:25:5,v/v/v)	20.0 mM of [BMIM][BF_4_]	[29]
Octopamine, synephrine and tyramine	Aqueous solution at pH 4.0	32 mM of [EMIM][BF_4_]	[30]
Norephedrine, ephedrine, pseudoephedrine and methylephedrine	Aqueous solution at pH 3.0	5.2-20.8 mM of [BMIM][BF_4_]	[31]
Norepinephrine, epinephrine, dopamine	Aqueous solution at pH 3.0	25 mM of [BMIM][BF_4_]	[32]
Neuroleptic drugs	NaH_2_PO_4_/ acetonitrile (60:40,v/v)	30.0 mM of [BMIM][PF_6_]	[33]
Fluoroquinolone antibiotics	10 mM ammonium acetate/acetonitrile(87:13,v/v)	5.0 mM of [BMIM][BF_4_]	[34]
Heterocyclic aromatic amines	Acetonitrile/water (19:81, v/v)	1.0 mM of [BMIM][BF_4_]	[35]
Seven β-blockers	Acetonitrile/water (30:70, v/v)	6.0 mM of [BMIM][BF_4_]	[36]
Guanine and hypoxanthine	Methanol /water (40:60, v/v) at pH 3.0	2.0 mM of [OMIM][MS]	[37]

**Table 3:** Extraction of organic and metal compounds from environmental contaminant.

Source	Target compounds	Ionic liquid	Extraction method	Ref.
Tap, bottled, fountain, well, river, rainwater, treated and raw wastewater	18 polycyclic aromatic hydrocarbons	[OMIM][PF_6_]	DLLME	[43]
Human urine	Methamphetamine, amphetamine	[EeMIM][NTf_2_]	SPME	[44]
Snow, river and brook water.	Four aromatic amines	[HMIM][PF_6_]	USA-DLLME	[45]
Tap, lake and fountain water	Fipronil, Chlorfenapyr, Buprofezin, and Hexythiazox	[HMIM][PF_6_]	DLLME	[46]
Tap, well, rain and Yellow River water	Organophosphorus pesticides	[OMIM][PF_6_]	DLLME	[47]
Everglade, river, reservoir and snow	Dichlorodiphenyltrichloroethane and its metabolites	[HMIM][PF_6_]	DLLME	[48]
Water and milk	Zinc	[HPy][PF_6_]	DLLME	[49]
NIST SRM 1643e, NIST SRM 1549	Lead	[BMIM][PF_6_]	SDME	[50]
Tap, well, river, and creek water	13 aromatic compounds	[BMIM][NTf_2_]	DLLME	[51]
Tap, well, Changjiang river, East lake, rain water	Cadmium	[HMIM][PF_6_]	USA-DLLME	[52]
Human urine	Seven phenothiazines derivatives	[BMIM][PF6]	LLE	[53]
East Lake and Yangtze River	Lovastatin and simvastatin	[HMIM][PF_6_]	USA-DLLME	[54]
Tap, river, underground water	Lead and nickel	[HMIM][PF_6_]	HF-LPME	[55]
Tap and lake water from South Lake	Chromium(VI)	[HMIM][PF_6_]	DLLME	[56]
Tap water, well water and rain	1-Naphthylamine, *N,N*-Dimethylaniline,Diphenylamine	[BMIM][PF_6_]	LLE	[57]
Human whole blood	Hemoglobin	[BTMSIM][PF_6_]	LLE	[58]
Storm water	Aliphatic and aromatic hydrocarbons	[BMIM][PF_6_]	HFM-LLLME	[59]
Sediments	Polycyclic aromatic hydrocarbons	[HDMIM][Br]	MAE	[60]
Furfural and acetic acid aqueous solution	Furfural	[HMIM][PF_6_]	LLE	[61]
East Lake and waste water	Phenols	[OMIM][PF_6_]	SDME	[62]
River water and effluentwater	Chlorobenzenes	[BMIM][PF_6_]	SDME	[63]
Taihu Lake	Inorganic mercury	[BTMSIM][PF_6_]	LLE	[64]

**Table 4:** Extraction of bioactive compounds from natural plant.

Source	Target compounds	Ionic liquid	Extraction Method	Ref.
*Psidium guajava Linn.* leaves and *Smilax china* tubers	Polyphenolic compounds	[BMIM]Br	MAE	[77]
*Stephaniae tetrandrae*	Fangchinoline, tetrandrine	[BMIM][BF_4_]	UAE	[78]
lotus leaf	*N*-nornuciferine, *O*-nornuciferine, nuciferine	[HMIM]Br	MAE	[79]
White pepper	Piperine	[BMIM][BF_4_]	UAE	[80]
Fruits of *Illicium verum Hook. f.* and *Cuminum cyminum L.*	Essential oils	[BMIM]PF_6_	MAE	[81]
Mixed Tocopherol	Tocopherol Homologues	[BMIM]Cl	LLE	[82]
*Nelumbo nucifera Gaertn*	Phenolic alkaloids	[BMIM][BF_4_]	MAE	[83]
-	Ferulic acid, caffeic acid	[BMIM][PF_6_]	LLE	[84]
*Lycoris Radiata*	Lycorine, lycoramine, galanthamine	[BMIM] CI	MMAE	[85]
*Rhizma Polygoni Cuspidati*	*trans*-Resveratrol	[BMIM]Br	MAE	[86]
Pea plants	3-Indole butyric acid	[BMIM][PF_6_]	LLE	[87]
Table grape and plum samples	Thiophanate-methyl, carbofuran,carbaryl, iprodione, hexythiazox, fenazaquin	[HMIM][PF_6_]	DLLME	[88]
Chilli powder, chilli oil and food additive	Para Red and Sudan dyes	[OMIM][PF_6_]	LE	[89]

**Table 5:** Application of ionic liquid as surface-bonded stationary phases.

Target compounds	Ionic liquids	Application	Ref.
Alcohols and aromatic compounds	[BMIM]Cl, [BMIM][NTf_2_]	GC stationary phases	[92]
Fatty acid methyl esters	1,9-di(3-vinyl-imidazolium)nonane bis(trifluoromethyl)sulfonylimidate	GC stationary phases	[93]
Acidic, basic, neutral compounds	1-octyl-3-propylimidazolium chloride	IL-based silica sorbent for SPE	[94]
Tanshinones	1-methylimidazole		[95]
Liquiritin, glycyrrhizic acid	2-ethyl-4-methylimidazole		[96]
Caffeine, theophylline theobromine	Imidazole, 1-methylimidazole, 2-ethyl-4-methylimidazole	IL-based silica sorbent as HPLC stationary phase	[97]
Ephedrines	[AHIm][BF_4_]		[98]
Peptides	Butyl-imidazol		[99]
Inorganic anions	Imidazolium functionalized silica		[100]
Caffeine, theophylline	1-methylimidazole	IL-based polymer sorbent for SPE	[101]
Esters	Bis[(trifluoromethyl)sulfonyl]imide		[102]

**Table 6:** Use of SILMs in gas and organic compounds separation.

Source	IL membrane	Application	Ref.
Steam reforming/water gas	[CnMIM][Tf_2_N]	Separating CO_2_ from H_2_	[111]
CO_2_/CH_4_ gas mixture	[C_3_NH_2_MIM][Tf_2_N], [C_3_NH_2_mim][CF_3_SO_3_]	Transport of CO_2_	[112]
CO_2_, N_2_, and CH_4_	[EMIM][dca]	Separation t of CO_2_	[113]
CO_2_, O_2_, N_2_ and CH_4_	[HMIM][Tf_2_N], [OMIM][Tf_2_N]	Separation t of CO_2_	[114]
CO_2_, N_2_, and CH_4_	[EMIM][Tf_2_N]	Separation of CO_2_	[115]
CO_2_/CH_4_ gas mixture	[BEIM][PF_6_]	Separation of CO_2_	[116]
Biohydrogen	[C_3_NH_2_MIM][CF_3_SO_3_]	Separation of biohydrogen	[117]
Penicillin G	TOMAC	Concentrating penicillin G	[118]
Acetone and butan-1-ol	[EEIM][PF_6_]	Separation of ternary mixtures	[119]
Chlorophenols	[OMIM][PF_6_]	Purification of water	[120]
Acidic gases	[BMIM][BF_4_]	Get rid of the acidic gases	[121]
Organic isomeric amines	[BMIM][PF_6_]	Selective separation	[122]
Organic acids, such as 4-phenoxybutyric acid, 3-phenoxypropionic acid and so on	[BMIM][PF_6_]	Lipase-facilitated transport	[123]
